# Cigarette smoking is a risk factor for the onset of fatty liver disease in nondrinkers: A longitudinal cohort study

**DOI:** 10.1371/journal.pone.0195147

**Published:** 2018-04-17

**Authors:** Masashi Okamoto, Teruki Miyake, Kohichiro Kitai, Shinya Furukawa, Shin Yamamoto, Hidenori Senba, Sayaka Kanzaki, Akiko Deguchi, Mitsuhito Koizumi, Toru Ishihara, Hiroaki Miyaoka, Osamu Yoshida, Masashi Hirooka, Teru Kumagi, Masanori Abe, Bunzo Matsuura, Yoichi Hiasa

**Affiliations:** 1 Department of Gastroenterology and Metabology, Ehime University Graduate School of Medicine, Shitsukawa, Toon, Ehime, Japan; 2 Ehime General Health Care Association, Misake, Matsuyama, Ehime, Japan; 3 Department of Epidemiology and Preventive Medicine, Ehime University Graduate School of Medicine, Shitsukawa, Toon, Ehime, Japan; 4 Epidemiology and Medical Statistics Unit, Translational Research Center, Ehime University Hospital, Shitsukawa, Toon, Ehime, Japan; 5 Department of Lifestyle-related Medicine and Endocrinology, Ehime University Graduate School of Medicine, Shitsukawa, Toon, Ehime, Japan; 6 Department of Internal Medicine, Saiseikai Matsuyama Hospital, Yamanishi, Matsuyama, Ehime, Japan; 7 Department of Community Medicine, Ehime University Graduate School of Medicine, Shitsukawa, Toon, Ehime, Japan; University College London, UNITED KINGDOM

## Abstract

**Background:**

The effect of cigarette smoking on the onset of nonalcoholic fatty liver disease (NAFLD) is unclear, especially that associated with drinking small amounts of alcohol. We conducted a longitudinal study to investigate the relationship between cigarette smoking and NAFLD onset, which was stratified according to the amount of alcohol consumed.

**Methods:**

We enrolled 7,905 Japanese subjects who had received annual health checkups more than twice between April 2003 and August 2013, 4,045 of whom met at least one of the following exclusion criteria and were excluded: (a) fatty liver at baseline; (b) hepatitis B or hepatitis C; (c) alcohol consumption (men: ≥210 g/wk; women: ≥140 g/wk); (d) change in alcohol drinking status between baseline and the study’s endpoint; (e) change in cigarette smoking habits between baseline and the study’s endpoint; or (f) current treatment with antidiabetic agents, antihypertensive agents, and/or lipid-lowering agents. The remaining 3,860 subjects (1,512 men, 2,348 women) were divided into two groups based on average alcohol consumption.

**Results:**

After adjusting for the variables associated with metabolic disease, smoking was associated with fatty liver disease onset compared with nonsmokers in nondrinkers (adjusted hazard ratio = 1.988, 95% confidence interval 1.057–3.595; *p* = 0.034). No association was found between smoking and fatty liver disease onset in the low alcohol consumption group (men: <210 g alcohol/week; women: <140 g alcohol/week). The fatty liver disease incidence increased significantly among the nondrinkers as the number of cigarettes smoked increased (*p* = 0.001).

**Conclusions:**

Cigarette smoking may be a significant risk factor associated with NAFLD onset in nondrinkers. These results may help clinicians to identify patients who are at a high risk of developing NAFLD and to prevent the progression of NAFLD by promoting earlier interventions that help people discontinue unhealthy lifestyle habits.

## Introduction

Nonalcoholic fatty liver disease (NAFLD) is a common lifestyle-related disease [1–3] that may progress to nonalcoholic steatohepatitis, cirrhosis, and liver failure [4, 5], and it may be a risk factor for the onset of type 2 diabetes and cardiovascular disease [6, 7]. Therefore, to prevent disease progression, it is important to identify patients who are at a high risk of NAFLD and to intervene with regard to lifestyle-related behaviors as early as possible.

Numerous studies have examined the relationships between cigarette smoking and lifestyle-related diseases, including type 2 diabetes, dyslipidemia, hypertension, and cardiovascular disease, and these studies’ findings have demonstrated that these pathological conditions are affected by cigarette smoking [8–10]. Similarly, cross-sectional studies’ findings have shown associations between NAFLD and cigarette smoking [11–14]. Liu et al. examined whether active and passive cigarette smoking were associated with NAFLD in a community-based survey that comprised 8,580 Chinese individuals who were aged ≥40 years, and they showed that both passive and heavy active cigarette smoking were associated with NAFLD [11]. However, Chavez-Tapia et al. investigated 933 healthy subjects who underwent annual health checkups at a university hospital in Mexico City, and they did not find an association between the prevalence of NAFLD and current or heavy cigarette smoking, which was defined as ≥20 packs/year, thereby suggesting that there was no relationship between cigarette smoking and NAFLD [14]. A longitudinal study investigated the effect of cigarette smoking on the onset of NAFLD [15], but the study included subjects whose smoking habits changed during the observation period. Therefore, the association between NAFLD and cigarette smoking remains unclear and somewhat controversial. Moreover, moderate alcohol intakes enhance insulin sensitivity [16], and are likely to prevent the onset of metabolic diseases, including fatty liver disease [17].

To clarify the association between cigarette smoking and the onset of NAFLD, it may be appropriate to categorize subjects according to the amounts of alcohol they consume. Therefore, we conducted a longitudinal cohort study to assess the epidemiologic relationship between cigarette smoking and the development of NAFLD in subjects who consumed low amounts of or no alcohol at baseline, and to analyze this relationship in all of the subjects, regardless of the amount of alcohol consumed.

## Methods

### Patients

We enrolled 7,905 subjects, comprising 3,863 men and 4,042 women whose ages ranged from 18 years to 80 years, who had undergone more than two annual health checkups between April 2003 and August 2013 at the Ehime General Health Care Association (S1 Data).

Before their health checkups, all of the subjects were asked to complete a questionnaire that gathered baseline data about their medical histories, prescribed medications, exercise habits (categorized as no exercise habit, conscious exercise or periodic exercise), snacking habits (categorized as no snacking, snacking less than once or more than twice per day), sleep durations (categorized as <5 h, ≥5–<7 h, ≥7– <9 h, or ≥9 h per night), alcohol consumption (frequencies and quantities), and the numbers of cigarettes smoked (categorized as 0, 1–14, 15–24, or ≥25 cigarettes per day) (S1 and S2 Questionnaire). Men with alcohol intakes of ≥210 g/week and women with alcohol intakes of ≥140 g/week were defined as alcohol drinkers [18].

The data analyzed also included the data from physical examinations and the results from routine biochemistry tests. The body mass index (BMI) was calculated based on a subject’s height and weight that were measured while the subjects wore light clothing and no shoes. Blood pressure was measured using an automated sphygmomanometer while the subjects were seated. Blood samples were collected after the subjects had fasted for ≥10 h, and the total cholesterol, triacylglycerol, fasting plasma glucose, uric acid, serum creatinine, hepatitis B surface antigen, and hepatitis C antibody levels were measured. Trained technicians who were blinded to each subject’s data, diagnosed fatty liver disease using abdominal ultrasonography (US) (Hitachi Vision Avius; Hitachi, Ltd. Tokyo, Japan). Fatty liver disease was diagnosed based on the following established diagnostic criteria: hepatorenal echo contrast, liver brightness, deep attenuation, and vascular blurring [19].

**Fig 1:**
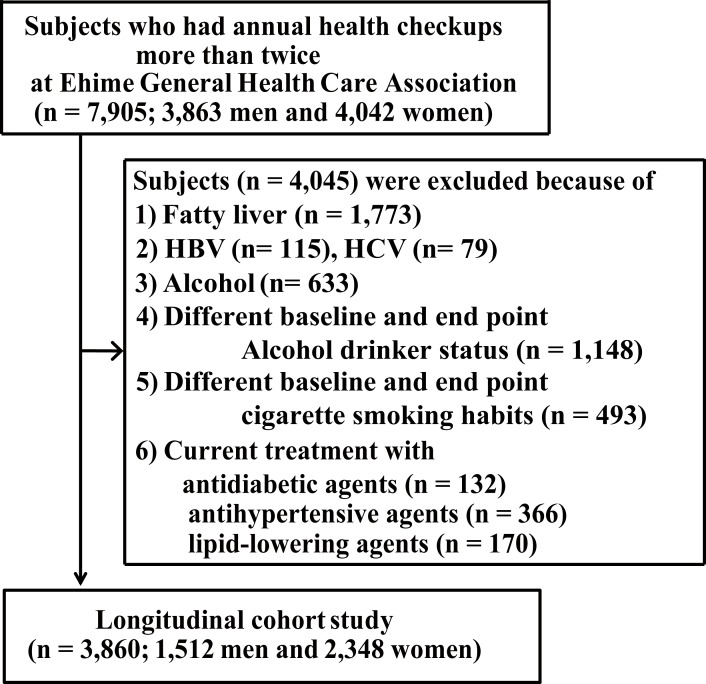
Flow chart of subject selection. Abbreviations: HBV, hepatitis B virus; HCV, hepatitis C virus.

Of the 7,905 subjects enrolled, 3,482 were excluded, because fatty liver disease was identified at baseline using abdominal US (*n* = 1,773), they tested positive for the hepatitis B surface antigen (*n* = 115) or the hepatitis C antibody (*n* = 79), their average alcohol consumption was ≥210 g/week if they were male or ≥140 g/week if they were female (*n* = 633), their alcohol consumption status changed from baseline to the study’s endpoint (*n* = 1,148), namely, from being a nondrinker to consuming low amounts of alcohol or vice versa, their cigarette smoking habit differed between baseline and the study’s endpoint (*n* = 493), that is, those who had newly stopped smoking or had newly started smoking, or because they were currently being treated with antidiabetic (*n* = 132), antihypertensive (*n* = 366), or lipid-lowering (*n* = 170) agents (Fig 1). After excluding these subjects, the medical records of 3,860 subjects, comprising 1,512 men and 2,348 women, were analyzed at Ehime University Hospital. The subjects who had never smoked (never-smokers) or had stopped smoking before baseline (ex-smokers) were classified as nonsmokers at baseline, and those who smoked cigarettes at baseline were classified as current smokers. In addition, the subjects were separated into two groups, namely, the nondrinker group and the low alcohol intake group that comprised men who drank <210 g alcohol/week and women who drank <140 g alcohol/week, based on their average alcohol consumptions (S1 and S2 Tables).

All of the participants were assigned a numerical code for the study period, and all of the data were stored on a secure database to maintain the subjects’ anonymity. Since this study was undertaken retrospectively, the requirement for informed consent to be obtained from the participants to enable them to participate in this study was waived. This study was conducted with a priori approval from Ehime University Hospital’s Research Ethics Board in accordance with the 1975 Declaration of Helsinki (Approval ID #110405, University Hospital Medical Information Network ID: UMIN000011953), and it fulfilled all local ethical and legal requirements. All of the procedures were performed in accordance with the Good Clinical Practice guidelines.

### Statistical analyses

The statistical analyses were performed using JMP software for Windows, version 12 (SAS Institute Inc., Cary, NC, USA). The continuous variables are presented as the medians and the interquartile ranges that comprise the 25^th^ and 75^th^ percentiles. The assumption of normality was analyzed using the Shapiro-Wilk test or the Kolmogorov-Smirnov-Lilliefors test, because the continuous variables were non-normally distributed. The Mann-Whitney U test was used to analyze the between-group differences in the baseline characteristics, which included age, the BMI, the blood pressure, and the biochemical variables derived from the routine blood tests. The categorical variables, including gender, the subjects’ baseline lifestyle habits, and the onset of fatty liver disease, are expressed as numbers and percentages, and they were analyzed using the chi-squared test. Univariate Cox proportional hazards regression analyses that used forward likelihood ratio tests were performed to evaluate the importance of each variable in relation to the incidence of fatty liver disease onset. The multivariate Cox proportional hazards regression analyses, which calculated the adjusted hazard ratios of the incidence of fatty liver disease associated with cigarette smoking, were adjusted with the following variables that are associated with metabolic disease: age, gender, the BMI, the systolic blood pressure, the total cholesterol, triacylglycerol, fasting plasma glucose, uric acid, and creatinine levels, exercise and snacking habits, and the sleep duration (Model 1). Alcohol consumption was additionally included as an adjustment to Model 1 to analyze the data irrespective of the amount of alcohol consumed. Model 2 included all of the factors that were found to be significant at *p* < 0.05 in the univariate analyses. The Cochran-Armitage test was used to assess the trends in the incidence rates of fatty liver disease based on numbers of cigarettes smoked. The Kaplan-Meier method was used to plot the fatty liver disease onset curves, and they were compared using log-rank tests. All of the reported *p* values are two-tailed, and a value of *p* < 0.05 was considered statistically significant.

## Results

### Baseline characteristics of the current smokers and nonsmokers

The mean observation period was 3.96 (standard deviation 2.5) years and the median observation period was 3.61 (range 0.44–10.13) years. Data were available from all of the subjects for all of the variables examined, except for the one subject’s BMI (0.023%). During the observation period, 443 subjects met the criteria for fatty liver disease onset. Tables 1 and 2 compare the smokers’ and nonsmokers’ characteristics according to the amount of alcohol consumed. Compared with the nonsmokers, the smokers were significantly more likely to be male, have a higher BMI, have higher levels of the biochemical parameters, for example, triglycerides, uric acid, and creatinine, and to snack, regardless of their alcohol consumption. The fatty liver disease incident rate was significantly higher in the smokers compared with that in the nonsmokers. The current smokers who consumed low levels of alcohol were older, and had higher systolic blood pressure and fasting plasma glucose levels, and the current smokers who did not consume alcohol had shorter sleep durations.

**Table 1:** Baseline characteristics of and the fatty liver disease incidence rate in the nondrinkers.

Total	Current smokers	Nonsmokers	*p* value
(*n* = 801)	(*n* = 68)	(*n* = 733)	
Age, years	44.5 (36.3–52)	42 (36–51)	0.36
Male gender, *n* (%)	49 (72.1)	91 (12.4)	<0.001
Body mass index, kg/m^2^	21.9 (20.4–23.4)	20.7 (19.2–22.5)	0.002
Systolic blood pressure, mmHg	102.5 (95–115)	107 (97.5–116)	0.112
Total cholesterol, mM	5.3 (4.6–5.8)	5.3 (4.7–6.0)	0.302
Triacylglycerols, mM	1.1 (0.9–1.5)	0.8 (0.6–1.1)	<0.001
Fasting plasma glucose, mM	5.1 (4.8–5.4)	4.9 (4.7–5.2)	0.019
Uric acid, μM	315.2 (249.8–350.9)	255.8 (220.1–297.4)	<0.001
Creatinine, μM	70.7 (61.9–79.6)	53.0 (53.0–61.9)	<0.001
*Exercise habit			
Periodic exercise, *n* (%)	17 (25.0)	211 (28.8)	0.508
**Snacking habit, *n* (%)			
Snacking	39 (57.4)	618 (84.3)	<0.001
***Sleep duration, *n* (%)			
Short sleep duration	45 (66.2)	429 (58.5)	0.220
Onset of fatty liver disease, *n* (%)	14 (20.6)	62 (8.5)	0.0011

*Exercise habit: no habit or conscious exercise vs. periodic exercise.

**Snacking habit: no snacking vs. snacking less than once or more than twice a day.

***Sleep duration: short sleep duration of ≤4 or 5–6 h vs. an adequate sleep duration of approximately 7–8 or ≥9 h.

The values are expressed as the medians and interquartile ranges (25th and 75th percentiles).

The differences between the groups in relation to the continuous variables were assessed using the Mann-Whitney U test. The chi-squared test was used to compare prevalence.

**Table 2:** Baseline characteristics of and the incidence rate of fatty liver in the low alcohol consumption group.

Total	Current smokers	Nonsmokers	*p* value
(*n* = 3,059)	(*n* = 492)	(*n* = 2,567)	
Age, years	41 (35–49)	40 (34–47)	0.002
Male gender, *n* (%)	438 (89.0)	934 (36.8)	<0.001
Body mass index, kg/m^2^	22.3 (20.5–24.4)	21.3 (19.7–23.1)	<0.001
Systolic blood pressure, mmHg	110 (101–120)	107 (98–118)	0.003
Total cholesterol, mM	5.1 (4.5–5.7)	5.1 (4.6–5.7)	0.358
Triacylglycerols, mM	1.1 (0.8–1.6)	0.8 (0.6–1.1)	<0.001
Fasting plasma glucose, mM	5.2 (4.9–5.4)	5.0 (4.7–5.3)	<0.001
Uric acid, μM	345.0 (297.4–386.6)	280.0 (232.0–339.0)	<0.001
Creatinine, μM	70.7 (70.7–79.6)	61.9 (53.0–70.7)	<0.001
*Exercise habit, *n* (%)			
Periodic exercise	147 (29.9)	839 (32.7)	0.223
**Snacking habit, *n* (%)			
Snacking	199 (40.5)	1,845 (71.9)	<0.001
***Sleep duration, *n* (%)			
Short sleep duration	295 (60.0)	1,575 (61.4)	0.561
Onset of fatty liver disease, *n* (%)	100 (20.3)	267 (10.4)	<0.001

*Exercise habit: no habit or conscious exercise vs. periodic exercise.

**Snacking habit: no snacking vs. snacking less than once or more than twice a day.

***Sleep duration: short sleep duration of ≤4 h or 5–6 h vs. an adequate sleep duration of approximately 7–8 h or ≥9 h.

The values are expressed as the medians and interquartile ranges (25th and 75th percentiles)The differences between the groups in relation to the continuous variables were assessed using the Mann-Whitney U test. The chi-squared test was used to compare prevalence.

### Baseline lifestyle characteristics of the nondrinker and the low alcohol consumption groups

A comparison of the nondrinker and low alcohol consumption groups with respect to the baseline lifestyle characteristics is shown in the S3 Table. Snacking was significantly more frequent in the nondrinker group compared with that in the low alcohol consumption group. The groups did not differ in relation to exercise or a short sleep duration.

### Risk factors for NAFLD onset in the nondrinker and low alcohol consumption groups

Tables 3 and 4 show the associations between the onset of fatty liver disease and a variety of parameters according to the amount of alcohol consumed. The univariate analyses showed that the current smokers had a significantly higher risk of fatty liver disease onset, regardless of the amount of alcohol consumed. In addition, age, being male, the BMI, the systolic blood pressure, the total cholesterol, triacylglycerol, fasting plasma glucose, uric acid, and creatinine levels, and snacking were significant factors associated with the risk of fatty liver disease development in both groups.

**Table 3:** Univariate analysis of the risk factors associated with the onset of fatty liver disease in the nondrinker group.

	HR (95% CI)	*p* value
Age	1.039 (1.014–1.065)	0.002
Male gender	4.081 (2.574–6.412)	<0.001
Body mass index	1.355 (1.279–1.432)	<0.001
Systolic blood pressure	1.024 (1.011–1.036)	<0.001
Total cholesterol level	1.339 (1.063–1.674)	0.014
Triacylglycerol level	2.607 (1.913–3.453)	<0.001
Fasting plasma glucose level	2.677 (1.772–3.900)	<0.001
Uric acid level	1.010 (1.007–1.013)	<0.001
Creatinine level	1.038 (1.022–1.053)	<0.001
*Exercise habit	0.773 (0.477–1.296)	0.319
**Snacking habit	0.505 (0.308–0.864)	0.014
***Sleep duration	1.324 (0.835–2.146)	0.236
Smoking status	3.596 (1.93–6.242)	<0.001

*Exercise habit: no habit or conscious exercise vs. periodic exercise.

**Snacking habit: no snacking vs. snacking less than once or more than twice per day.

***Sleep duration: short sleep duration of ≤4 h or 5–6 h vs. an adequate sleep duration of approximately 7–8 h or ≥9 h.

Abbreviations: HR, hazard ratio, CI confidence interval.

**Table 4:** Univariate analysis of the risk factors associated with the onset of fatty liver disease in the low alcohol consumption group.

	HR (95% CI)	*p* value
Age	1.037 (1.025–1.049)	<0.001
Male gender	2.639 (2.13–3.287)	<0.001
Body mass index	1.309 (1.277–1.342)	<0.001
Systolic blood pressure	1.022 (1.016–1.028)	<0.001
Total cholesterol level	1.547 (1.383–1.728)	<0.001
Triacylglycerol level	1.678 (1.57–1.781)	<0.001
Fasting plasma glucose level	1.302 (1.195–1.396)	<0.001
Uric acid level	1.006 (1.005–1.008)	<0.001
Creatinine level	1.018 (1.012–1.023)	<0.001
*Exercise habit	0.952 (0.767–1.188)	0.661
**Snacking habit	0.720 (0.585–0.889)	0.002
***Sleep duration	1.026 (0.834–1.265)	0.809
Smoking status	2.069 (1.637–2.594)	<0.001

*Exercise habits: no habit or conscious exercise vs. periodic exercise.

**Snacking habit: no snacking vs. snacking less than once or more than twice per day.

***Sleep duration: short sleep duration of ≤4 h or 5–6 h vs. an adequate sleep duration of approximately 7–8 h or ≥9 h.

Abbreviations: HR, hazard ratio, CI confidence interval.

### Association between cigarette smoking and the onset of fatty liver disease

The variables found to be associated with metabolic disease were included in the multivariate analyses. Model 1 included all of the parameters investigated, namely, age, gender, the BMI, the systolic blood pressure, the total cholesterol, triacylglycerol, fasting plasma glucose, uric acid, and creatinine levels, the exercise and snacking habits, and the sleep duration, while Model 2 included the parameters that were significant in the univariate analyses, namely, age, being male, the BMI, the systolic blood pressure, the total cholesterol, triacylglycerol, fasting plasma glucose, uric acid, and creatinine levels, and snacking. A significant association was found between cigarette smoking and the onset of fatty liver disease in the nondrinker group (Table 5).

**Table 5:** Association between cigarette smoking and the onset of fatty liver disease.

	Nondrinker group		Low alcohol consumption group	
	aHR (95% CI)	*p* value	aHR (95% CI)	*p* value
Model 1	2.162 (1.065–4.195)	0.034	1.144 (0.88–1.478)	0.311
Model 2	2.246 (1.099–4.379)	0.027	1.165 (0.9–1.5)	0.243

Model 1 was adjusted for all of the variables that were associated with metabolic disease, namely, age, gender, the body mass index, the systolic blood pressure, the total cholesterol, triacylglycerol, fasting plasma glucose, uric acid, and creatinine levels, the exercise and snacking habits, and the sleep duration. Model 2 was adjusted for all of the factors that were significant in the univariate analyses.

Abbreviations: aHR, adjusted hazard ratio; CI confidence interval.

### Association between the number of cigarettes smoked and fatty liver disease onset in the nondrinker group

**Fig 2:**
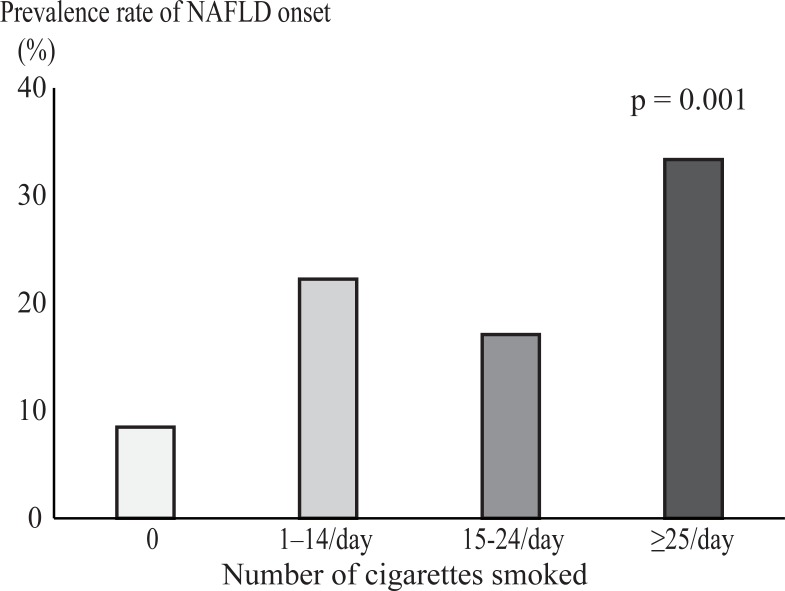
Association between the number of cigarettes smoked and the incidence of fatty liver disease in the nondrinker group. The *p* values were determined using the Cochran-Armitage test. NAFLD, nonalcoholic fatty liver disease.

An investigation into the relationship between the number of cigarettes smoked and fatty liver disease onset in the nondrinker group showed that the incidence of fatty liver disease increased significantly as the number of cigarettes smoked increased (*p* = 0.001) (Fig 2).

### Risk factors associated with fatty liver disease onset in all of the subjects

The current smokers had a significantly higher risk of fatty liver disease onset based on the univariate analyses (S4 Table). In addition, age, being male, the BMI, the systolic blood pressure, the total cholesterol, triacylglycerol, fasting plasma glucose, uric acid, and creatinine levels, snacking, and a low level of alcohol consumption were significantly associated with the risk of developing fatty liver disease (S4 Table).

### Association between cigarette smoking and fatty liver disease onset in all of the subjects

Model 1 included all of the parameters, namely, age, gender, the BMI, the systolic blood pressure, the total cholesterol, triacylglycerol, fasting plasma glucose, uric acid, and creatinine levels, exercise and snacking habits, and the sleep duration, while Model 2 included those parameters that were significant in the univariate analyses, namely, age, being male, the BMI, the systolic blood pressure, the total cholesterol, triacylglycerol, fasting plasma glucose, uric acid, and creatinine levels, snacking, and the consumption of low levels of alcohol. A significant association was found between smoking and fatty liver disease onset in Model 2 (S5 Table).

### Incidence of fatty liver disease according to the subjects’ smoking habits

We divided the subjects into the smoker and nonsmoker groups to evaluate the incidence of fatty liver disease in the two groups. The cumulative incidence of NAFLD was significantly higher in the smoker group than that in the nonsmoker group (*p* < 0.001). In the smoker and nonsmoker groups, the 1-year fatty liver disease onset rates were 3% and 1.4%, respectively, the 3-year fatty liver disease onset rates were 12.1% and 6.2%, respectively, the 5-year fatty liver disease onset rates were 22.9% and 11.6%, respectively, and the 10-year fatty liver disease onset rates were 48.4% and 20.1%, respectively (S1 Fig).

## Discussion

The findings from this longitudinal study, which excluded subjects whose smoking habits changed during the observation period, demonstrated that cigarette smoking was a significant risk factor associated with the onset of fatty liver disease in individuals who did not consume alcohol, and that this significant association persisted after adjusting for the potential confounders. Moreover, the incidence of fatty liver disease in the nondrinker group increased significantly as the number of cigarettes smoked increased. However, cigarette smoking was not a risk factor for the onset of fatty liver disease in subjects who consumed low amounts of alcohol.

In a previous longitudinal cohort study, Hamabe et al. enrolled 1,560 subjects who had undergone complete medical health checkups in 1998 and 2008, which included abdominal US at the Kagoshima Kouseiren Medical Healthcare Center, to examine the association between smoking and the development of NAFLD [15]. The multivariate analysis that was adjusted for age, gender, the BMI, hypertension, dyslipidemia, dysglycemia, and the alcohol intake, showed that cigarette smoking (odds ratio [OR] = 1.91, 95% confidence interval [CI] 1.34–2.72) was a risk factor associated with the development of NAFLD, and that the subjects’ smoking status at baseline was associated with the development of NAFLD (Brinkman index: 1–399; OR = 1.77, 95% CI 1.02–3.07 and Brinkman index: ≥400; OR = 2.04, 95% CI 1.37–3.03). However, this association was not significant in subjects excluding former smokers, new smokers, and new quitters (OR = 1.64, 95% CI 0.99–2.72) [15]. The authors thought that the study’s results may have been affected by the modest sample size and increases in the BMIs of the subjects who had recently stopped smoking. Therefore, the relationship between cigarette smoking and the onset of NAFLD remains unclear. Furthermore, the participants in the present study were separated based on the amounts of alcohol they consumed. Previous studies’ results have shown that the frequency of cigarette smoking increases as the amount of alcohol consumed increases [20]. In addition, the findings from several studies have shown that the consumption of moderate levels of alcohol decreases the risk of fatty liver disease onset [17, 21]. Low levels of alcohol consumption improve insulin sensitivity [16], enhance hepatic blood flow [22], and increase the adiponectin levels [16, 23, 24]; these effects might support our finding that cigarette smoking accompanied by low intakes of alcohol is not a risk factor for the onset of fatty liver disease.

Several mechanisms underlie the effect of cigarette smoking on the development of NAFLD. Yuan et al. showed that cigarette smoke stimulated the accumulation of lipids in hepatocytes in apoB100 transgenic mice and in cultured cells that inactivated adenosine monophosphate-activated kinase, which, in turn, contributed to the increased activation of sterol regulatory element binding protein-1 [25]. In addition, cigarette smoking has been shown to be associated with insulin resistance [26]. Azzalini et al. examined obese Zucker rats that were exposed to firsthand cigarette smoke via a nose-only system, and they found that cigarette smoke induced insulin resistance and oxidative stress, which led to hepatic steatosis and exacerbated hepatocellular ballooning and lobular inflammation [26]. Therefore, cigarette smoking might adversely affect the pathophysiology of NAFLD.

The primary strengths of the present study relate to the recruitment of the participants from the general population and the completeness of the data for all of the relevant variables; indeed, only one datum was missing. In addition, none of the subjects’ smoking habits changed during the observation period. Finally, the subjects were stratified based on the amount of alcohol they consumed, which enabled the effect of cigarette smoking on fatty liver disease onset to be examined in the context of the effect of alcohol consumption. On the other hand, our study had several limitations. First, we only collected data annually; hence, our data were not truly continuous. Second, several factors relied on information that was self-reported; therefore, misreported data could have distorted our findings. Third, we did not examine the effects of the duration of cigarette smoking on fatty liver disease onset. The observation period differed for each subject in this study, and we did not examine the smoking durations of the former smokers sufficiently; therefore, we did not examine the effect of the smoking duration on fatty liver disease onset adequately. Forth, we did not examine the type of alcohol consumed. Fifth, we did not distinguish between NAFLD and nonalcoholic steatohepatitis, and we were unable to examine the severity of the NAFLD. Finally, we only evaluated Japanese subjects; therefore, other populations should be examined to corroborate the generalization of our results.

Despite these limitations, our study’s results showed that cigarette smoking was a significant risk factor for the onset of fatty liver disease in the participants who did not consume alcohol. Moreover, the incidence of fatty liver disease among the subjects who did not consume alcohol increased as the number of cigarettes smoked increased. Taken together with the findings from previous studies, our observations support recommendations to cease smoking to help prevent the onset of fatty liver disease. These results may help clinicians to identify patients who are at a high risk of developing NAFLD and to prevent the progression of NAFLD by promoting earlier interventions that help people discontinue unhealthy lifestyle habits.

## Supporting information

S1 Table Categories based on average alcohol consumptions (Men). (DOC)

S2 Table Categories based on average alcohol consumptions (Women). (DOC)

S3 Table Baseline lifestyle characteristics of the nondrinker and the low alcohol consumption groups. (DOCX)

S4 Table Univariate analyses of the risk factors associated with the onset of fatty liver disease in all of the subjects. (DOCX)

S5 Table Association between cigarette smoking and the onset of fatty liver disease in all of the subjects. (DOCX)

S1 Fig Kaplan-Meier curves of the cumulative onset rate of fatty liver disease onset based on the subjects’ smoking habits. Kaplan-Meier curves of the event-free durations according to the subjects’ smoking habits. In the smoker and nonsmoker groups, the 1-year fatty liver disease onset rates were 3% and 1.4%, respectively, the 3-year fatty liver disease onset rates were 12.1% and 6.2%, respectively, the 5-year fatty liver disease onset rates were 22.9% and 11.6%, respectively, and the 10-year fatty liver disease onset rates were 48.4% and 20.1%, respectively. The cumulative onset rate of fatty liver disease was significantly higher in the smokers compared with that in the nonsmokers (*p* < 0.001).
(EPS)

S1 Questionnaire (In Japanese). (PDF)

S2 Questionnaire (Translated into English). (DOCX)

S1 Data Raw data of the entire subjects (N = 7,905). (XLSX)
