# Supplementary material for: Cigarette smoking is a risk factor for the onset of fatty liver disease in nondrinkers: A longitudinal cohort study
Source: PLoS One. 2018 Apr 17;13(4):e0195147. doi: 10.1371/journal.pone.0195147 (PMC5903610; doi:10.1371/journal.pone.0195147)
Supplement: S2 Table — (DOC) [file pone.0195147.s002.doc]

**S2 Table. Categories based on average alcohol consumptions (Women)**

|  |  | **Quantity of daily alcohol consumption in average** | | | | |
| --- | --- | --- | --- | --- | --- | --- |
|  |  | **None** | **Less than 20g** | **More than 20g and less than 40g** | **More than 40g and less than 60g** | **More than 60g** |
| **Frequency of alcohol consumption in average** | **Occasionally (between several times per year and less than once a week)** | nondrinker group | low alcohol intake group | | | |
|  | **Once or twice a week** |  |  |  |  |  |
|  | **Between 3 times and 5 times a week** |  |  |  | alcohol drinker group | |
|  | **Almost every day** |  |  |  |  |  |
