# Supplementary material for: Cigarette smoking is a risk factor for the onset of fatty liver disease in nondrinkers: A longitudinal cohort study
Source: PLoS One. 2018 Apr 17;13(4):e0195147. doi: 10.1371/journal.pone.0195147 (PMC5903610; doi:10.1371/journal.pone.0195147)
Supplement: S3 Table — (DOCX) [file pone.0195147.s003.docx]

**S3 Table. Baseline lifestyle characteristics of the nondrinker and the low alcohol consumption groups.**

| Total (*n* = 3,860) | Nondrinker group  (*n* = 801) | Low alcohol consumption group  (*n* = 3,059) | *p* value |
| --- | --- | --- | --- |
| *Exercise habits, *n* (%)  Periodic exercise | 228 (28.5) | 986 (32.3) | 0.409 |
| **Snacking habit, *n* (%)  Snacking | 657 (82.0) | 2,044 (66.8) | <0.001 |
| ***Sleep duration, *n* (%)  Short sleep duration | 474 (59.2) | 1,870 (61.1) | 0.313 |

*Exercise habit: no habit or conscious exercise vs. periodic exercise.

**Snacking habit: no snacking vs. snacking less than once or more than twice per day.

***Sleep duration: short sleep duration of ≤4 h or 5–6 h vs. an adequate sleep duration of approximately 7–8 h or ≥9 h.

The chi-squared test was used to compare the prevalence.
