# Supplementary material for: Cigarette smoking is a risk factor for the onset of fatty liver disease in nondrinkers: A longitudinal cohort study
Source: PLoS One. 2018 Apr 17;13(4):e0195147. doi: 10.1371/journal.pone.0195147 (PMC5903610; doi:10.1371/journal.pone.0195147)
Supplement: S4 Table — (DOCX) [file pone.0195147.s004.docx]

**S4 Table. Univariate analyses of the risk factors associated with the onset of fatty liver disease in all of the subjects.**

|  | HR (95% CI) | *p* value |
| --- | --- | --- |
| Age | 1.036 (1.025–1.047) | <0.001 |
| Male gender | 2.815 (2.325–3.418) | <0.001 |
| Body mass index | 1.317 (1.288–1.347) | <0.001 |
| Systolic blood pressure | 1.023 (1.017–1.028) | <0.001 |
| Total cholesterol level | 1.477 (1.337–1.629) | <0.001 |
| Triacylglycerols level | 1.712 (1.608–1.812) | <0.001 |
| Fasting plasma glucose level | 1.336 (1.237–1.424) | <0.001 |
| Uric acid level | 1.007 (1.006–1.008) | <0.001 |
| Creatinine level | 1.020 (1.015–1.025) | <0.001 |
| *Exercise habit | 0.912 (0.748–1.116) | 0.367 |
| **Snacking habit | 1.490 (1.227–1.803) | <0.001 |
| ***Sleep duration | 1.072 (0.887–1.298) | 0.474 |
| Smoking status | 2.245 (1.807–2.769) | <0.001 |
| Alcohol | 1.286 (1.011–1.658) | 0.04 |

*Exercise habit: no habit or conscious exercise vs. periodic exercise.

**Snacking habit: no snacking vs. snacking less than once or more than twice per day.

***Sleep duration: short sleep duration of ≤4 h or 5–6 h vs. an adequate sleep duration of approximately 7–8 h or ≥9 h.

Abbreviations: HR, hazard ratio, CI confidence interval.
