# Supplementary material for: Cigarette smoking is a risk factor for the onset of fatty liver disease in nondrinkers: A longitudinal cohort study
Source: PLoS One. 2018 Apr 17;13(4):e0195147. doi: 10.1371/journal.pone.0195147 (PMC5903610; doi:10.1371/journal.pone.0195147)
Supplement: S5 Table — (DOCX) [file pone.0195147.s005.docx]

**S5 Table. Association between cigarette smoking and fatty liver disease onset in all of the subjects.**

|  | aHR (95% CI) | *p* value |
| --- | --- | --- |
| Model 1 | 1.24 (0.971–1.575) | 0.084 |
| Model 2 | 1.274 (1.000–1.614) | 0.0497 |

Model 1 was adjusted for all of the variables that were associated with metabolic disease, namely, age, gender, the body mass index , the systolic blood pressure, the total cholesterol, triacylglycerols, fasting plasma glucose, uric acid, and creatinine levels, the exercise and snacking habits, the sleep duration, and the alcohol consumption status. Model 2 was adjusted for all of the factors that were significant in univariate analyses.

Abbreviations: aHR, adjusted hazard ratio; CI, confidence interval.
