# Supplementary material for: Cigarette smoking is a risk factor for the onset of fatty liver disease in nondrinkers: A longitudinal cohort study
Source: PLoS One. 2018 Apr 17;13(4):e0195147. doi: 10.1371/journal.pone.0195147 (PMC5903610; doi:10.1371/journal.pone.0195147)
Supplement: S1 Questionnaire — (PDF) [file pone.0195147.s007.pdf]

1）平均睡眠時間

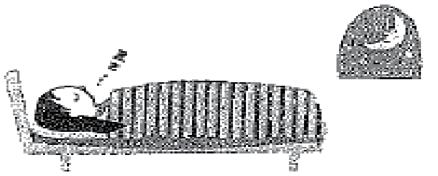

①９時間以上
②７～９時間未満
③５～７時間未満
④５時間未満

3）間食について
（果物は除く）

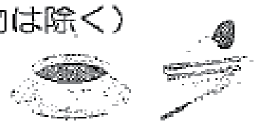

①ほとんどとらない
②１日１回以内
③１日２回以上

4）飲酒について

この質問でのお酒1合とは、純アルコール量に換算して20g程度を示します。（180mlに相当するとは限りません）

ビール（５％）なら
中瓶１本（500ml）程度

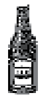

焼酎（25％）なら
ストレートで（100ml）程度

日本酒（15％）なら
１合（180ml）程度

ウイスキー（40％）なら
ダブル１杯（60ml）程度

ワイン（11％）なら
ワイングラス２杯（240ml）程度

①まったく飲まない
②１合未満
③１～２合未満
④２～３合未満
⑤３合以上

● 頻度　②～⑤の方のみ
①たまに　１年に数回～月３回
②週に１～２回
③週に３～５回
④ほぼ毎日

5）喫煙について

①まったく吸わない（もしくは禁煙10年以上）
②現在禁煙している（10年未満）

※左のイラストをご参考にお答えください。
③習慣的に吸っている
④習慣的には吸っていない

②～④の方のみ
● 一日の喫煙本数

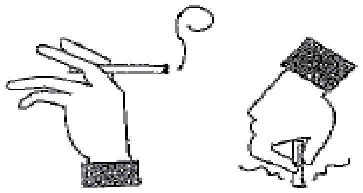

①約10本（目安１～14本くらい）
②約20本（目安15～24本くらい）
③約30本（目安25本以上）

7）運動習慣について　※左のイラストをご参考にお答えください。

①定期的な運動習慣がある
② ①以外の方で日常生活で体をよく動かすよう努力している
③特に体を動かすよう心がけていない
