# Supplementary material for: Cigarette smoking is a risk factor for the onset of fatty liver disease in nondrinkers: A longitudinal cohort study
Source: PLoS One. 2018 Apr 17;13(4):e0195147. doi: 10.1371/journal.pone.0195147 (PMC5903610; doi:10.1371/journal.pone.0195147)
Supplement: S2 Questionnaire — (DOCX) [file pone.0195147.s008.docx]

**S2 Questionnaire**

Answer to the questions below.

Q. Daily hours of sleep in average

More than 9 hours

More than 7 hours and no more than 9 hours

More than 5 hours and no more than 7 hours

No more than 5 hours

Q. Snack habit

None or almost none

Once a day

More than twice a day

Q. Alcohol consumption: quantity in average (see Appendix below)

None

No more than 20g per day

More than 20g and no more than 40g per day

More than 40g and no more than 60g per day

More than 60g per day

Appendix

500ml of beer, 100ml of Japanese distilled liquor, 180ml of Japanese sake, 60ml of whiskey and 240ml of wine are considered as 20g of pure alcohol intake.

Q. Alcohol consumption: frequency in average

Occasionally (between several times per year and no more than once a week)

Once or twice a week

Between 3 times and 5 times a week

Almost every day

Q. Smoking habit

Never or none for more than 10 years

Quitted smoking within 10 years

Current smoker (occasional smoker)

Current smoker (daily smoker)

Q. Quantity of daily smoking

Approximately 10 (between 1 and 14)

Approximately 20 (between 15 and 24)

Approximately 30 (more than 25)

Q. Exercise habit

Periodic exercise

Conscious exercise

No exercise habit
